# Revisiting the Era of Intestinal Tuberculosis: A Case Presenting As Small Bowel Obstruction With Classical Imaging and Histopathological Appearances

**DOI:** 10.7759/cureus.51836

**Published:** 2024-01-08

**Authors:** Kajal Hatgoankar, Anand Hatgaonkar, Pratibha Dawande

**Affiliations:** 1 Pathology, Datta Meghe Medical College, Datta Meghe Institute of Higher Education and Research, Nagpur, IND; 2 Radiodiagnosis, Datta Meghe Medical College, Datta Meghe Institute of Higher Education and Research, Nagpur, IND

**Keywords:** computed tomography, intestinal stricture, small-bowel obstruction, intestinal tuberculosis, extrapulmonary tuberculosis (eptb)

## Abstract

The incidence of tuberculosis (TB) worldwide is still significantly high, with India contributing a high global TB burden. This case study features a 49-year-old male who had complaints of pain and abdominal distention for one and a half months. An erect abdominal radiograph showed features suggesting small bowel obstruction. Contrast-enhanced computed tomography (CT) of the abdomen was done. It showed multiple strictures involving the distal jejunum and ileum, causing small bowel obstruction. There was mesenteric and retroperitoneal lymphadenopathy with central necrosis and ascites. The patient was operated on for a small bowel obstruction. The resected intestine showed four strictures, tiny nodules on the serosal surface, and many enlarged lymph nodes. Representative tissue from these areas showed the typical picture of multiple caseating granulomas and fibrosis. Ziehl-Neelsen (ZN) staining highlighted the acid-fast bacilli (AFB). The suspicion index for intestinal tuberculosis (ITB) should be kept high while evaluating patients with intestinal obstruction presenting in endemic areas and high-risk populations, such as HIV-infected, undernourished, immunocompromised, and those with diabetes, smoking, and alcohol addiction.

## Introduction

Tuberculosis (TB) is a very old enemy of humankind. There is scientific evidence of the presence of this disease in the human population since ancient times [1]. It has always been one of the leading infectious causes of morbidity and mortality. In recent years, coronavirus disease (COVID-19) has exceeded it. In 2021, the incidence of TB was around 1.6 million worldwide. About 1.6 million people died from the disease [2]. India was among the eight countries on the list of high-burden countries (HBC), accounting for more than two-thirds of the global burden of TB. India’s contribution to TB-related death in the human immunodeficiency virus (HIV)-infected population was 36% globally [2].

The underdiagnosis of TB cases is a real issue in India. The chances of developing TB are much higher in HIV-infected patients, in patients with diabetes, and in people addicted to alcohol and smoking. Malnourished and immunosuppressed people are especially at increased risk of acquiring the disease. TB primarily affects the lungs; however, it can affect any organ of the body when it is called extrapulmonary tuberculosis (EPTB). EPTB accounts for more than 40% of TB in HIV-positive individuals and about 10-20% in immunocompetent patients [3]. Due to this association, HIV testing is recommended for any individual diagnosed with EPTB. Despite the various advances in the diagnosis and treatment of TB, we still encounter many cases of TB, especially EPTB, in our daily practice. This article highlights how, with a high level of suspicion, we can catch this disease in its early stages and avoid complications related to advanced disease with prompt and early treatment.

## Case presentation

**Figure 1:**
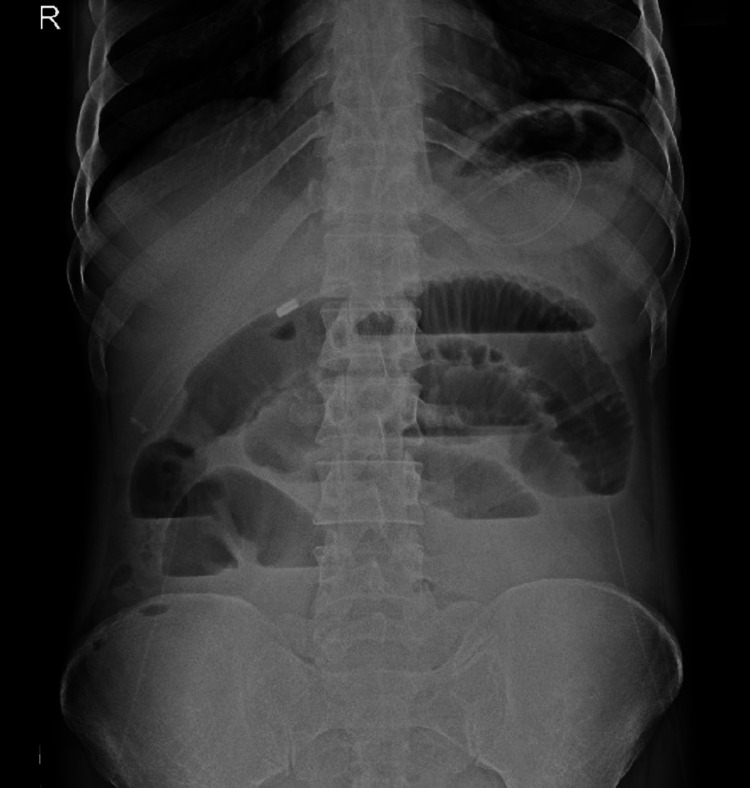
Radiograph of the abdomen in standing view The erect abdominal X-ray revealed dilated small bowel loops with multiple air-fluid levels, suggestive of small bowel obstruction.

A 49-year-old man was admitted to a tertiary care hospital serving a rural population in central India. He complained of abdominal pain and abdominal distension for one and a half months. He also complained of constipation for the last couple of months. On examination, the abdomen was rigid, distended, and tender. Guarding of the abdomen was present. An erect abdominal radiograph revealed dilated loops of the small intestine with multiple air-fluid levels suggestive of small intestine obstruction (Figure 1).

**Figure 2:**
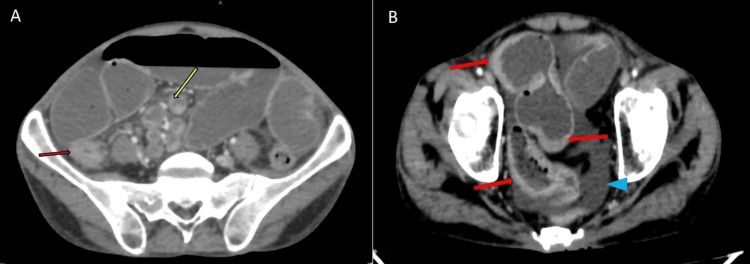
Contrast computed tomography scan of the abdomen, axial views. Image A: A contrast computed tomography scan axial image showing abdominal lymphadenopathy (yellow arrow) and small bowel stricture (red arrow). Image B: Contrast computed tomography scan axial image showing long segment circumferential bowel wall thickening with luminal narrowing suggesting small bowel stricture (red arrows) and ascites (blue arrowhead).

**Figure 3:**
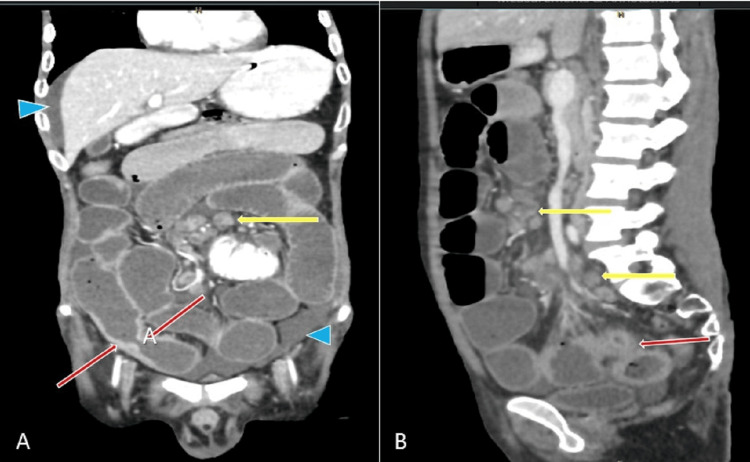
Contrast computed tomography scan, coronal and sagittal views Image A: Contrast computed tomography scan coronal image showing abdominal lymphadenopathy (yellow arrow), small bowel stricture (red arrows), and ascites (blue arrowheads). Image B: Contrast computed tomography scan sagittal image showing abdominal lymphadenopathy (yellow arrows) and small bowel stricture (red arrow).

A contrast computed tomography (CT) scan of the abdomen was performed. It showed multiple strictures involving the distal jejunum and ileum, causing small intestine obstruction. Mesenteric and retroperitoneal lymphadenopathy with central necrosis was observed along with mild to moderate ascites (Figures 2, 3).

**Figure 4:**
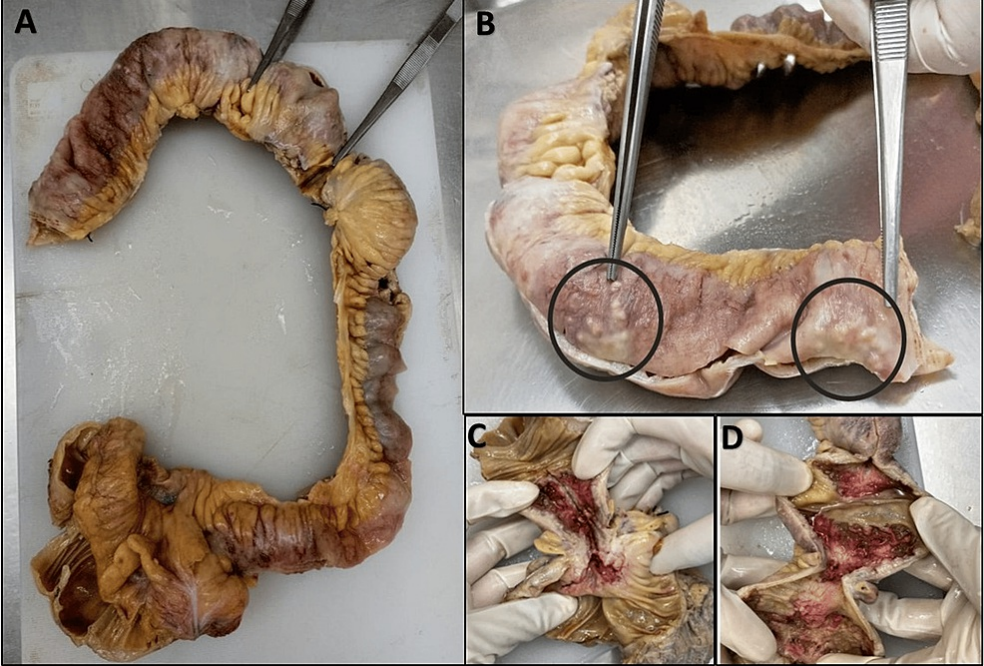
Gross appearance of resected bowel Image A: Specimen of resected bowel showing areas of strictures (pointed by forceps). Image B: Subserosal nodules/tubercles (highlighted by black circles). Images C and D: Cut sections of the intestine from the areas of strictures showing the bowel wall thickening.

The radiological findings favored an infectious or inflammatory etiology with a more likely possibility of intestinal TB than Crohn’s disease. Clinical and histopathological correlation was recommended. The patient underwent surgery for acute small bowel obstruction. Intraoperative findings of multiple strictures in the ileum and jejunum were observed, as well as many enlarged lymph nodes. We received a 62 cm long specimen of the resected bowel, which contained the jejunum, ileum, and caecum with a small part of the ascending colon. A total of four strictures were identified. The serosal surface had many tiny nodules/tubercles (Figure 4). A total of 22 lymph nodes were identified.

**Figure 5:**
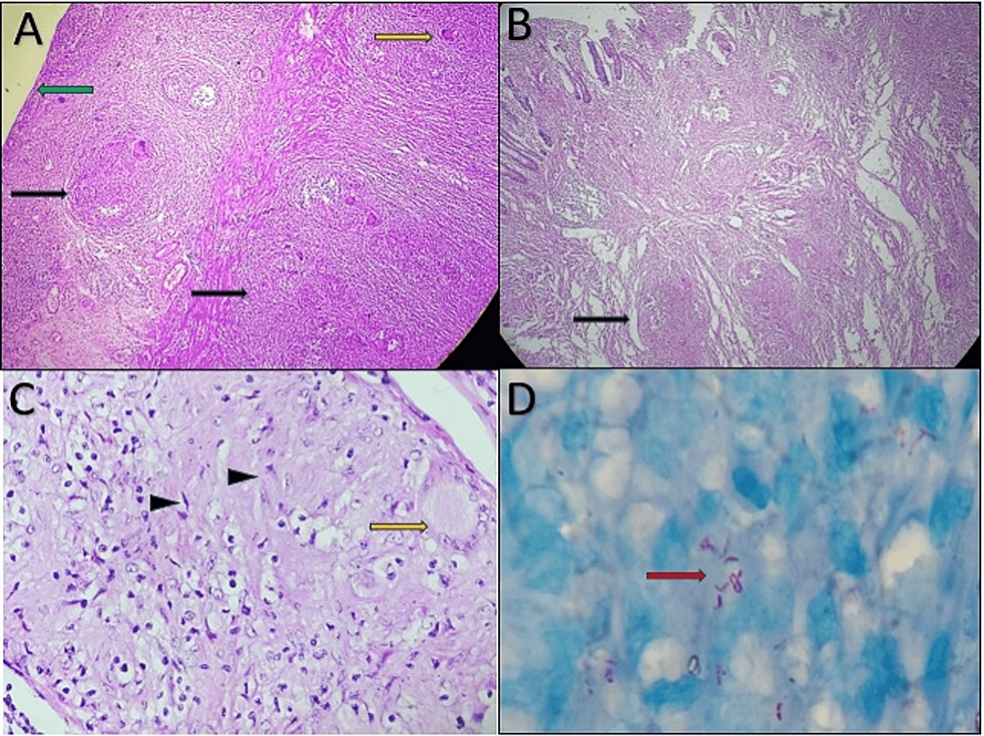
Histomicrograph of the intestinal strictured areas and lymph node Image A: Granuloma (black arrows) with multinucleated giant cells (yellow arrow) are present in all layers of the intestine - mucosa, muscularis propria, extending up to serosa (Hematoxylin and Eosin stain, 100x). Image B: Subserosal nodule (black arrow) showing granulomas (Hematoxylin and Eosin stain, 100x). Image C: The lymph node has been replaced by granulomas, characterized by epithelioid cells (black arrowheads) (Hematoxylin and Eosin stain, 400x). Image D: Ziehl-Neelsen stain of subserosal nodule showing acid-fast bacilli (red arrow) (1000x).

Microscopy revealed mucosal ulceration at the site of the stricture. Multiple caseating granulomas were identified spread across the mucosa, submucosa extending to serosa along with extensive fibrosis. The Ziehl-Neelsen (ZN) staining highlighted acid-fast bacilli (AFB). Fourteen out of the 22 lymph nodes showed granulomatous reaction and ZN staining did not reveal any AFB (Figure 5).

A diagnosis of intestinal tuberculosis (ITB) was made. The patient has started a six-month regimen of isoniazid (H), rifampicin (R), ethambutol (E), and pyrazinamide (Z), all four drugs for the first two months, followed by H and R for the remaining four months.

## Discussion

India has always been one of the top candidates when it comes to TB. Even with the advent of very effective therapy and many government policies such as the National TB Elimination Program (NTEP) and Pradhan Mantri TB Mukt Bharat Abhiyaan (PMTBMBA) to provide treatment and additional nutritional, diagnostic, and vocational support to patients affected by TB and their families, the situation has not improved as expected [4]. The morbidity, mortality, and cost of health care associated with this disease are still very high.

Although pulmonary TB is the most common form of TB in the community, there has been an increase in EPTB due to HIV. According to the India TB Report 2023, around 24% of the patients had EPTB. Common sites of EPTB were the lymph node (26.3%), pleural (23.3%), abdomen (17.4%), spine (4.8%), meninges (2.8%), bone (excluding the spine; 2.7%), and genitourinary tract (1.6%) [4].

Gastrointestinal tuberculosis (GITB) can be primary (the site of the initial infection being the intestine) or secondary (disseminated). The ways by which the bacilli reach the gastrointestinal tract are (1) ingestion of tubercular bacilli in sputum from pulmonary TB; (2) lymphatic or hematogenous spread from a primary focus (lung); (3) direct spread from adjacent organs; (4) reactivation of latent TB [5-7]; and (5) ingestion of milk products contaminated with *Mycobacterium bovis* [8-10]. In our case, we were unable to determine the source of infection or spread to the intestine.

The clinical presentation of intestinal TB is very nonspecific. They may include abdominal pain, vomiting, diarrhea, constipation, fever, anorexia, and weight loss [8]. Intestinal TB is not an uncommon cause of intestinal obstruction. The diagnosis of ITB is not always straightforward, especially when relying only on clinical findings. Even in endemic areas, the precision of clinical diagnosis is only 50% [11]. Patients with intestinal TB may not always have associated signs of pulmonary TB [12]. Therefore, delays in diagnosis are frequent, and subsequent delays in the initiation of treatment lead to high morbidity and mortality [8].

The presentation of GITB can be divided into three categories: hypertrophic form (10%), ulcerative form (60%), and ulcero-hypertrophic form presenting as a mass lesion (30%). These presentations are nonspecific and may be similar to other gastrointestinal conditions, such as peptic ulcer, Crohn’s disease, fungal infections, or malignancies, leading to misdiagnosis [13,14]. After ileocecal mass, stricture of the small intestine (22%) was the second most common surgical finding. The underlying pathology of stricture appears to be due to fibrosis associated with the granulomatous reaction, which is characteristic of tubercular infection anywhere in the body. The granulomatous involvement of the mesenteric vessels, in the form of intraluminal thrombi or perivascular cuffs, that leads to gut ischemia may also contribute to the development of strictures [15]. Lymph node involvement is common in GITB. The commonly involved lymph nodes are the mesenteric, omental, porta hepatis, celiac axis, and peripancreatic area lymph nodes [5].

Radiological techniques, such as X-ray, CT, or magnetic resonance imaging (MRI), are used to evaluate the extent of diseases. A contrast CT scan of the abdomen is the gold standard for the diagnosis of ITB, preferably performed with negative oral contrast, which can very well show thickening of the bowel wall and stricture formation leading to obstruction. The presence of ascites and necrotizing lymphadenopathy is well appreciated. Thorough knowledge of the key radiological characteristics of GITB can lead to early detection of the disease as well as timely intervention and treatment [16]. The histopathology of stricture shows the classical picture of multiple caseating granulomas spread across the mucosa and submucosa, extending to the serosa, along with extensive fibrosis. A similar picture is also seen in the enlarged conglomerated lymph nodes. ZN staining highlights the AFB.

## Conclusions

The case discussed here highlights the need to increase awareness of TB in general and extrapulmonary TB in particular. Nonspecific clinical symptoms and signs, mimicking some other gastrointestinal conditions, such as malignancy, may pose a significant challenge in the diagnosis of GITB. Hence, the diagnosis of GITB only on clinical grounds is rarely accurate. The suspicion index for ITB should be kept high while evaluating patients with intestinal obstruction who present in endemic areas and high-risk populations, such as HIV-infected, undernourished, immunocompromised, and those with diabetes and smoking and alcohol addiction. The primary care physician is likely to evaluate the initial presentation of EPTB. Therefore, they must be alert and aware of the changing epidemiological conditions and the varied presentation of the disease. An improved early diagnosis of TB is essential and is the cornerstone of minimizing morbidity and healthcare costs.
